# Suspected Pregabalin Overdose Presenting With Out-of-Hospital Asystolic Cardiac Arrest and Fatal Multiorgan Failure: A Case Report

**DOI:** 10.7759/cureus.115355

**Published:** 2026-08-28

**Authors:** Muhammed Baspinar

**Affiliations:** 1 Anesthesiology and Reanimation, Elazığ Fethi Sekin City Hospital, Elazığ, TUR

**Keywords:** drug intoxication, drug overdose, multiple organ dysfunction, post cardiac arrest care, pregabalin toxicity

## Abstract

Pregabalin overdose is generally considered to have a relatively benign clinical course. However, severe respiratory depression, coma, and life-threatening clinical deterioration have rarely been reported, particularly in patients with high-dose ingestion or delayed recognition. We present the case of a 26-year-old male who was found unconscious following suspected intentional ingestion of approximately 14 pregabalin 300 mg capsules (4.2 g).

On arrival of the emergency medical team, the patient was found to be in out-of-hospital cardiac arrest with an initial rhythm of asystole. Return of spontaneous circulation was achieved after 20 minutes of cardiopulmonary resuscitation. On admission, profound metabolic acidosis (pH: 6.43) and severe hyperlactatemia (lactate: 29 mmol/L) were present. During follow-up, the patient developed rhabdomyolysis, acute kidney injury requiring renal replacement therapy, marked transaminase elevation compatible with ischemic hepatitis, and pancreatic enzyme elevation. Despite aggressive supportive treatment, the patient died on the fourth day due to refractory shock and multiorgan failure.

Although pregabalin overdose is often benign, suspected high-dose ingestion may lead to profound central nervous system depression, loss of airway protective reflexes, aspiration, respiratory arrest, and fatal post-cardiac arrest multiorgan failure. Early airway protection and close hemodynamic monitoring are essential. We present a rare, fatal case of a young adult who developed multi-organ dysfunction syndrome following suspected severe pregabalin intoxication.

## Introduction

Pregabalin is a structural analogue of gamma-aminobutyric acid that binds to the α2​δ subunit of voltage-gated calcium channels and reduces excitatory neurotransmitter release [1]. It is commonly used for neuropathic pain, epilepsy, fibromyalgia, and generalized anxiety disorder [1]. Because of its widespread clinical use, pregabalin exposure and intentional overdose presentations have become increasingly relevant in emergency medicine, toxicology, and intensive care practice [2]. Pregabalin is predominantly eliminated unchanged by the kidneys, and clinically significant toxicity may be more pronounced in patients with impaired renal function or concomitant central nervous system depressant exposure [1].

Most pregabalin overdoses are reported to follow a mild or moderate clinical course [2,3]. In observational toxicology series, isolated pregabalin overdose usually presents with somnolence, dizziness, confusion, ataxia, and mild central nervous system depression [2,3]. In a dose-toxicity evaluation, higher pregabalin doses were associated with more severe poisoning, although large interindividual variability was observed [3]. Therefore, clinical risk assessment should not rely solely on the estimated ingested dose but should also include symptoms, comorbidities, co-exposures, respiratory status, and airway protection [3].

Pregabalin cannot be considered completely harmless in overdose. Regulatory safety communications have reported severe respiratory depression associated with pregabalin, including cases occurring without concomitant opioid use [4]. The risk of respiratory depression appears higher in patients with compromised respiratory function, neurological disease, renal impairment, older age, or concomitant use of central nervous system depressants [4]. Severe pregabalin poisoning requiring intensive care support and extracorporeal elimination has also been reported, although such cases remain uncommon [5,6].

Fatal outcomes after suspected pregabalin ingestion are clinically important because death may not result from direct cardiotoxicity alone. A plausible mechanism is profound central nervous system depression leading to impaired airway protective reflexes, possible aspiration, hypoventilation, respiratory arrest, hypoxic cardiac arrest, and post-cardiac arrest multiorgan failure. This mechanism is particularly relevant when patients are found unconscious outside the hospital, and the exact duration of hypoxia is uncertain. Here, we present a young male patient with suspected severe pregabalin intoxication who developed out-of-hospital asystolic cardiac arrest, profound acidosis, severe hyperlactatemia, rhabdomyolysis, acute kidney injury, ischemic hepatic and pancreatic injury, and fatal multiorgan failure.

The rationale for presenting this report is to highlight that suspected high-dose pregabalin exposure, even in young individuals without known comorbidities, warrants immediate airway-focused evaluation and pre-hospital vigilance. Furthermore, this report aims to remind emergency physicians and intensivists that the catastrophic multiorgan failure observed in such presentations stems primarily from severe post-resuscitation ischemic-reperfusion injury and prolonged tissue hypoxia rather than direct intrinsic drug organotoxicity.

## Case presentation

A 26-year-old male patient with no known chronic medical disease was found unconscious at his workplace and was evaluated by emergency medical services. According to information obtained from the scene, an empty blister package was found nearby, suggesting an unconfirmed, suspected ingestion of approximately 14 pregabalin 300 mg capsules (estimated total dose of 4.2 g). Neither quantitative serum pregabalin concentration measurement nor comprehensive toxicological screening could be performed because these tests were not available at our institution; blood alcohol testing was negative (<10 mg/dL). The suspected ingestion occurred between 13:30 and 14:00. The patient was last seen well at approximately 14:40, and emergency medical services were called at 14:45.

On the arrival of the emergency medical team, the patient was in out-of-hospital cardiac arrest with an initial rhythm of asystole. Endotracheal intubation and CPR were immediately initiated at the scene by the emergency medical services (EMS) team. Cardiopulmonary resuscitation was continued during pre-hospital transport, and return of spontaneous circulation (ROSC) was achieved upon arrival in the emergency department after approximately 20 minutes of cumulative resuscitation efforts.

Upon arrival in the ED, the patient was deeply comatose (Glasgow Coma Scale (GCS) score: 3) with fixed, dilated pupils. Initial post-ROSC vital signs demonstrated profound hypotension requiring immediate initiation of peripheral noradrenaline infusion. A bedside electrocardiogram revealed sinus tachycardia with non-specific ST-T changes. Arterial blood gas analysis confirmed profound mixed acidosis with severe lactic acid accumulation. Immediate point-of-care echocardiography revealed preserved left ventricular function without gross valvular pathology. Following initial stabilization and advanced diagnostic evaluation in the ED, the patient was transferred to the ICU.

**Figure 1:**
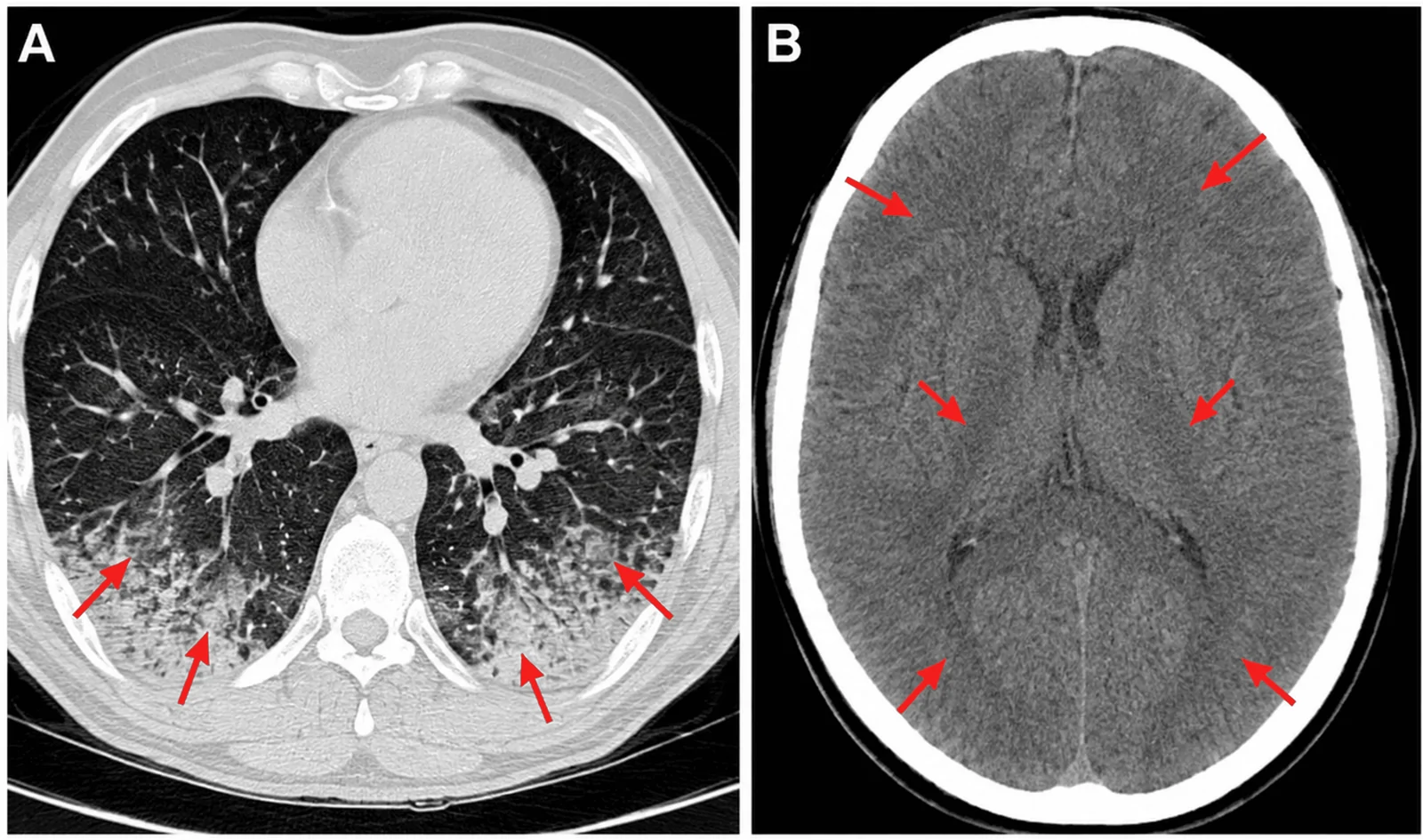
Thoracic and cranial CT A: Axial thoracic CT of the lung window demonstrates bilateral posterior-basal consolidative and ground-glass opacities (red arrows) in the dependent portions of both lower lobes, compatible with aspiration-related pulmonary involvement. B: Axial non-contrast cranial CT shows diffuse loss of gray-white matter differentiation, effacement of cortical sulci, and narrowing of the lateral ventricles (red arrows), findings suggestive of global hypoxic-ischemic cerebral injury following cardiopulmonary arrest.

On intensive care admission, the patient was deeply comatose and mechanically ventilated. Initial arterial blood gas analysis revealed profound mixed metabolic and respiratory acidosis, with a pH of 6.43, pCO₂ of 61 mmHg, and lactate level of 29 mmol/L. Thoracic CT demonstrated bilateral posterior-basal pulmonary opacities compatible with possible aspiration-related pulmonary involvement (Figure 1). Cranial imaging showed findings suggestive of hypoxic-ischemic cerebral injury following cardiopulmonary arrest (Figure 1). The clinical condition was therefore interpreted as suspected pregabalin-related central nervous system depression complicated by possible aspiration, respiratory arrest, out-of-hospital asystolic cardiac arrest, and post-cardiac arrest syndrome.

The diagnosis of pregabalin intoxication was considered probable rather than confirmed based on the presence of a pregabalin box at the scene, the estimated ingestion of 4.2 g pregabalin, the temporal relationship, and the compatible clinical course. However, we could not measure serum pregabalin concentration or perform comprehensive toxicological screening because these tests were not available at our institution.

During intensive care follow-up, the patient received advanced cardiac life support, endotracheal intubation, invasive mechanical ventilation, antimicrobial therapy, and high-dose vasopressor support. Laboratory findings showed progressive multiorgan dysfunction, including severe rhabdomyolysis, marked transaminase elevation compatible with ischemic hepatitis, coagulopathy, and pancreatic enzyme elevation. Continuous renal replacement therapy (CRRT) was initiated primarily for severe metabolic acidosis, acute kidney injury, and rhabdomyolysis in the context of post-cardiac arrest multiorgan failure (Table 1).

**Table 1:** Chronological laboratory and perfusion trends pCO_2_: Partial pressure of carbon dioxide

Parameter	Reference range	Day one	Day two	Day three	Day four
pH	7.35-7.45	6.43	6.92	7.01	7.1
Lactate (mmol/L)	0.5-2.2	29	12	11	8.5
Aspartate aminotransferase (U/L)	0-35	247	5084	6100	6036
Alanine aminotransferase (U/L)	0-35	432	3084	3800	3677
Amylase (U/L)	28-100	415	9269	7003	5655
Lipase (U/L)	13-60	239	2870	4792	11289
Creatinine (mg/dL)	0.7-1.2	1.2	4.36	5.32	2.07
pCO_2_ (mmHg)	35-45	61	55	52	44
Partial pressure of oxygen (pO2) (mmHg)	80-120	174	145	128	123
Blood alcohol concentration (mg/dL)	0-0.5	0	0	0	0

Despite aggressive supportive treatment, the patient developed persistent hypotension and progressive multiorgan failure. He died on the fourth day of intensive care follow-up after recurrent cardiopulmonary resuscitation. The final clinical diagnosis was suspected pregabalin overdose complicated by possible aspiration, out-of-hospital asystolic cardiac arrest, hypoxic-ischemic injury, rhabdomyolysis, acute kidney injury, ischemic hepatic/pancreatic injury, refractory shock, and fatal multiorgan failure.

## Discussion

Pregabalin is a gabapentinoid that binds to the α2​δ subunit of voltage-gated calcium channels and is widely used for neuropathic pain, epilepsy, fibromyalgia, and generalized anxiety disorder [1]. Its increasing clinical use has been accompanied by a growing number of intentional and recreational overdose presentations in emergency departments [2]. Most isolated pregabalin overdoses are generally reported to have a mild or moderate clinical course, usually presenting with somnolence, dizziness, confusion, ataxia, or mild central nervous system depression [2,3]. However, severe manifestations such as coma, seizures, respiratory depression, and the need for intensive care support have also been described, particularly in high-dose ingestions, delayed presentations, renal impairment, or co-ingestion with sedative agents [2-5].

In the present case, a young male patient without known chronic disease developed out-of-hospital asystolic cardiac arrest after suspected intentional ingestion of approximately 4.2 g pregabalin. Although this dose is lower than some previously reported non-fatal massive pregabalin ingestions, the rapid progression to unconsciousness, respiratory compromise, asystolic arrest, profound lactic acidosis, and fatal multiorgan failure makes the case clinically important. This observation supports the view that the severity of pregabalin intoxication should not be evaluated only by the estimated ingested dose but also by the time to recognition, airway protection, respiratory status, aspiration risk, and post-arrest physiology [3].

The most plausible mechanism in this case is not direct pregabalin-induced cardiotoxicity, but pregabalin-related central nervous system depression leading to impaired airway protective reflexes, possible aspiration, hypoventilation, respiratory arrest, and subsequent hypoxic asystolic cardiac arrest. Regulatory safety communications have warned that pregabalin may be associated with severe respiratory depression, including cases reported without concomitant opioid use [4]. In this patient, the absence of documented aspiration material limits certainty; however, thoracic imaging findings compatible with aspiration-related pulmonary involvement and the clinical sequence of unconsciousness followed by respiratory and cardiac arrest support this mechanism.

Following return of spontaneous circulation, the patient developed profound metabolic acidosis, severe hyperlactatemia, rhabdomyolysis, acute kidney injury, coagulopathy, marked transaminase elevation, pancreatic enzyme elevation, vasopressor-dependent shock, and progressive multiorgan failure. These findings are best interpreted as part of post-cardiac arrest syndrome and ischemic multiorgan injury rather than as direct organ-specific pregabalin toxicity. In particular, the marked elevation of transaminases and pancreatic enzymes was considered compatible with ischemic hepatic and pancreatic injury in the setting of prolonged hypoperfusion and refractory shock.

There is no specific pharmacological antidote available for pregabalin intoxication [1]. While empirical naloxone administration is frequently utilized in comatose overdose presentations in the emergency setting, it does not reverse pure gabapentinoid toxicity unless concurrent opioid co-ingestion is present [1,2]. Management remains strictly supportive, centered on early airway control, mechanical ventilation, and hemodynamic stabilization [1].

Renal replacement therapy was initiated because of severe metabolic acidosis, acute kidney injury, and rhabdomyolysis. Pregabalin is predominantly eliminated unchanged by the kidneys, and extracorporeal elimination may reduce pregabalin concentrations in selected severe cases [1,6]. The Extracorporeal Treatments In Poisoning (EXTRIP) Workgroup considers gabapentin and pregabalin dialyzable, particularly in patients with impaired kidney function; however, extracorporeal treatment is not routinely recommended in all gabapentinoid poisonings and should be individualized according to clinical severity, renal function, coma requiring mechanical ventilation, and associated complications [6]. In the present case, renal replacement therapy was clinically justified primarily by post-arrest metabolic derangement, acute kidney injury, and rhabdomyolysis rather than solely by pregabalin elimination.

A primary limitation of this report is the inability to analytically confirm serum pregabalin levels or exclude occult co-ingestions via comprehensive toxicological screening. Consequently, this presentation must be framed as suspected severe pregabalin intoxication rather than confirmed isolated poisoning. Alternative explanations, including the unrecognized co-ingestion of synthetic sedatives, illicit opioids, primary arrhythmic/cardiac arrest, or unmeasured metabolic derangements, cannot be definitively excluded in the pre-hospital phase. Nevertheless, the temporal proximity between the suspected ingestion, the discovery of empty blister packs, and the rapid deterioration supports a plausible clinical association.

This case has several clinically relevant implications. First, suspected high-dose pregabalin ingestion should not automatically be considered benign, even in young patients without known comorbidities. Second, altered consciousness after pregabalin ingestion should prompt early airway-focused assessment because loss of airway reflexes may lead to aspiration, respiratory arrest, and hypoxic cardiac arrest. Third, patients presenting after delayed recognition or prehospital collapse require aggressive post-arrest care and close monitoring for rhabdomyolysis, acute kidney injury, ischemic hepatitis, coagulopathy, and multiorgan failure. Finally, when available, obtain serum pregabalin concentration and broad toxicological screening early, as they substantially strengthen diagnostic certainty and publication quality.

## Conclusions

Pregabalin overdose is often considered to have a relatively benign course, but suspected high-dose ingestion may be associated with profound central nervous system depression, possible aspiration, respiratory arrest, out-of-hospital cardiac arrest, and fatal post-arrest multiorgan failure. This case emphasizes that clinicians should not rely solely on the estimated ingested dose when assessing pregabalin intoxication. Early airway protection, close respiratory monitoring, intensive care follow-up, and comprehensive toxicological evaluation are essential in patients with altered consciousness after suspected pregabalin overdose.
